# Talking to patients about their new spectacles

**Published:** 2024-05-15

**Authors:** Leah Kennan

**Affiliations:** 1 Ophthalmic nurse: City Eye Hospital City, Nairobi, Kenya.

**Educating patients, listening to their concerns, and offering guidance and reassurance will enhance their overall experience of wearing spectacles.**

**Figure:**
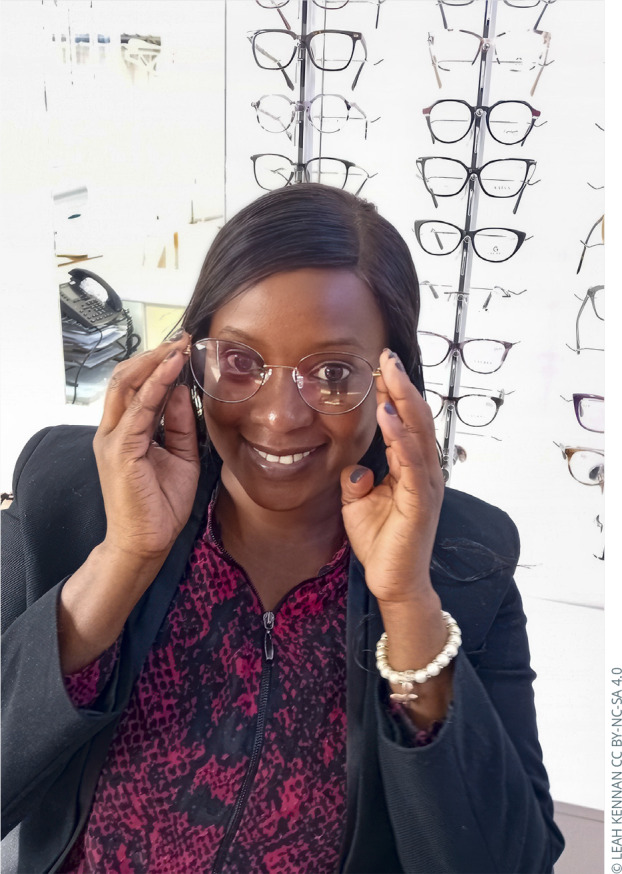
Put spectacles on and take them off using both hands to avoid tension on one side of the frame. kenya

Many people feel apprehensive when they are first told that they need a pair of spectacles. Here are some common examples we have come across, and ideas for addressing them.

- **Long-term use of spectacles.** Some patients feel if they wear spectacles for a long time their eyes may become sunken; reassure them that spectacles do not affect the anatomy of the eye.
- **Cosmetic appearance.** Some patients worry that the spectacles may not look good on their face; the person dispensing should provide a mirror to the patient while selecting the frames so they can reassure themselves about their appearance.
- **Effect of spectacles on vision.** Some patients are hesitant to wear spectacles, claiming that their vision might deteriorate. Assure them that the spectacles will improve their sight and that wearing corrective lenses will not make their vision worse in the long term.

## When dispensing spectacles

It is normal for people to experience some initial discomfort when they start wearing spectacles. Without the correct information and support, some patients may stop wearing their spectacles before they can benefit from the improvements in their vision the spectacles provide.

Therefore, when patients try on their new spectacles, take a moment to talk to them about the following (as appropriate):

- **Peripheral blurring due to the frame.** Some patients may experience blurring in their peripheral vision due to the edge of the frame. Reassure them that this effect becomes less noticeable over time
- **Depth perception.** Patients may experience temporary issues with depth perception whilst their eyes adjust to the new corrective lenses. Reassure them that they will adjust to this quickly.
- **Distortion.** This may be noticed more with strong prescriptions; patients may report a ‘fishbowl’ effect or other visual distortions which make them feel uncomfortable or disoriented, particularly if it's a new prescription or a different lens design. Again, assure them that this will becomes less noticeable over time, as they adjust.

## Before patients take their spectacles home

Ideally, patients should return for a check-up after one year. Explain to patients that they may need to come back sooner if they are unable to adjust to the prescription, if the spectacles break, or if they notice a change in their vision.

Some patients believe that spectacles ‘expire’ after a certain amount of time. Before the patient goes home, explain that the lens, when properly taken care of, will continue to work. Any changes in vision they experience may be due to scratches on the lens surface, or because there has been in a change in their vision.

Encourage patients to care for their spectacles (see panel) and to return for eye tests at regular intervals, depending on the nature of their condition. Remind patients to avoid sharing spectacles with relatives or friends as the prescription will be different and the user is likely to experience worsened vision and other symptoms, such as headaches or dizziness, as a result.

## Patients who need correction for both distance and near vision

Some patients may have specific visual requirements for both close work (such as reading or sewing) and distance vision (such as driving or seeing objects that are far away).

There are two main options for these patients:

1. Prescribing two (or more) separate pairs of spectacles – e.g., one for near vision and one for distance vision.
2. Prescribing bifocal (Figure 4) or progressive lenses – these provide two or more optical powers in a single lens, so that the patient has more than one working distance. Bifocal lenses have a visible horizontal line where the two lens powers meet; in progressive lenses there are no visible lines.

**Advice for patients: how to care for your spectacles**

By following these guidelines, your spectacles will last longer and the lenses will remain clear.

**Figure 1:**
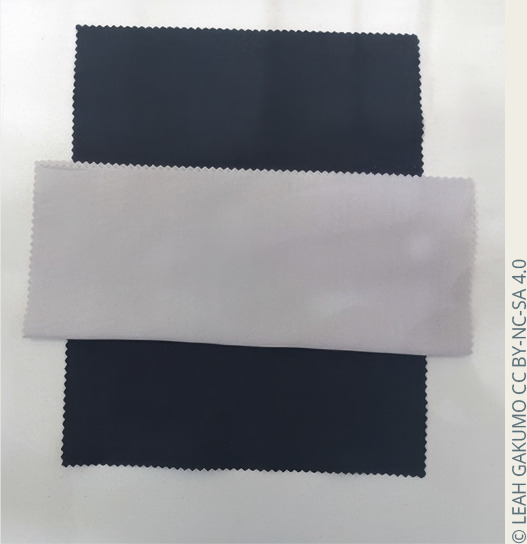
Use a microfibre cloth. Avoid using clothing, tissues, or paper towels as these can scratch the lens surface.

1. Use both hands when putting on or removing your spectacles to avoid bending the frames. The arm may also become loose on one side or may even break if taking on and off using just one hand.
2. Avoid harsh cleaners. Clean the lenses with mild soapy water or lens cleaner (specifically designed for spectacles) and dry them with a microfibre cloth (Figure 1). Avoid using clothing, tissues, or paper towels as these can scratch the lens surface.
3. Avoid exposing your spectacles to hairspray, perfumes, or other chemicals.
4. If your spectacles feel loose or uncomfortable, visit an optician who can adjust them. You can regularly check for any loose screws and tighten these gently, if needed.

**Storing spectacles**

**Figure 2:**
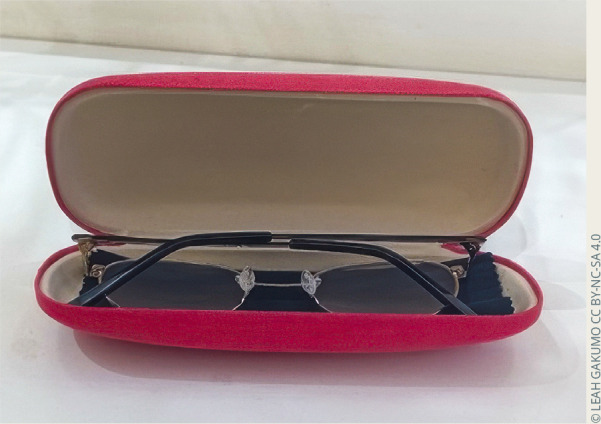
How to store spectacles in a case: lenses down, with the arms uppermost.

**Figure 3:**
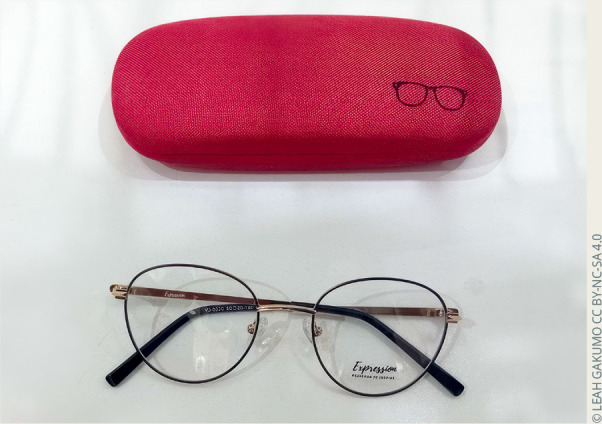
Place the spectacles on a surface with the lenses facing upwards.

1. Always store your spectacles in a hard-shell case for added protection. Ensure the lenses are lying on the microfibre cloth and the frame arms are facing upwards; this avoids damage to the arms (Figure 2).
2. Keep spectacles away from direct sunlight and extreme temperatures. For example, avoid placing them on a windowsill or on the dashboard of a moving vehicle; heat from the sun or the engine may damage the coating of the lenses and cause the frames to expand.
3. In case you need to place the spectacles on a surface without the storage case, ensure the arms of the frame are placed on the surface while the lenses are facing upward; this prevents the lenses from getting scratch marks (Figure 3).

**Advice for patients who need correction for both distance and near vision**

**How to use separate pairs of spectacles**

1. Wear your distance vision spectacles as advised by your practitioner, especially if they are needed for driving.
2. Only wear your near vision spectacles for near vision tasks such as reading, sewing, writing, drawing, cooking, or food preparation.

**How to use bifocal or progressive spectacles**

1. Look through the upper part of the spectacles to see objects in the distance, such as road signs.
2. Look through the lower part of the lens for tasks that require near vision, such as reading, sewing, writing, drawing, cooking, or food preparation.
3. If you have progressive lenses, look through the middle part of the lens to see objects in the middle distance, e.g., when working on a computer.
4. Take care when walking up or down steps or stairs. You may need to bend your head down so that you can look through the upper part the lenses. If you look through the lower part of the lenses, you may fall as the image may be blurred; the stairs may also appear nearer than they really are.

**Figure 4:**
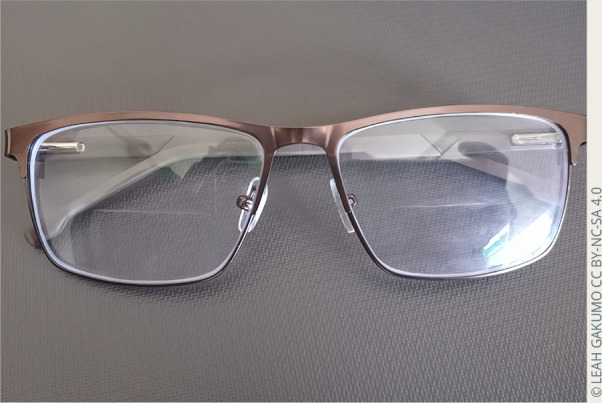
Bifocals

